# Characterization of periprosthetic environment microbiome in patients after total joint arthroplasty and its potential correlation with inflammation

**DOI:** 10.1186/s12879-023-08390-x

**Published:** 2023-06-22

**Authors:** Hao Li, Jun Fu, Niu Erlong, Rui LI, Chi Xu, Libo Hao, Jiying Chen, Wei Chai

**Affiliations:** 1 Medical School of Chinese PLA, Beijing, People’s Republic of China; 2 Department of Orthopedic Surgery, The First Medical Center, Chinese PLA General Hospital, 28 Fuxing Road, Beijing, People’s Republic of China; 3 Department of Orthopedics, 305 Hospital of PLA, Beijing, People’s Republic of China; 4 Senior Department of Orthopedics, Fourth Medical Center, Chinese PLA General Hospital, Beijing, People’s Republic of China

**Keywords:** Periprosthetic joint infection, Total joint arthroplasty, Metagenomic next generation sequencing, Microbiome, Microbiota, Polymicrobial infection

## Abstract

**Aims:**

Periprosthetic joint infection (PJI) is one of the most serious complications after total joint arthroplasty (TJA) but the characterization of the periprosthetic environment microbiome after TJA remains unknown. Here, we performed a prospective study based on metagenomic next-generation sequencing to explore the periprosthetic microbiota in patients with suspected PJI.

**Methods:**

We recruited 28 patients with culture-positive PJI, 14 patients with culture-negative PJI, and 35 patients without PJI, which was followed by joint aspiration, untargeted metagenomic next-generation sequencing (mNGS), and bioinformatics analysis. Our results showed that the periprosthetic environment microbiome was significantly different between the PJI group and the non-PJI group. Then, we built a “typing system” for the periprosthetic microbiota based on the RandomForest Model. After that, the ‘typing system’ was verified externally.

**Results:**

We found the periprosthetic microbiota can be classified into four types generally: “*Staphylococcus* type,” “*Pseudomonas* type,” “*Escherichia* type,” and “*Cutibacterium* type.” Importantly, these four types of microbiotas had different clinical signatures, and the patients with the former two microbiota types showed obvious inflammatory responses compared to the latter ones. Based on the 2014 Musculoskeletal Infection Society (MSIS) criteria, clinical PJI was more likely to be confirmed when the former two types were encountered. In addition, the *Staphylococcus* spp. with compositional changes were correlated with C-reactive protein levels, the erythrocyte sedimentation rate, and the synovial fluid white blood cell count and granulocyte percentage.

**Conclusions:**

Our study shed light on the characterization of the periprosthetic environment microbiome in patients after TJA. Based on the RandomForest model, we established a basic “typing system” for the microbiota in the periprosthetic environment. This work can provide a reference for future studies about the characterization of periprosthetic microbiota in periprosthetic joint infection patients.

**Supplementary Information:**

The online version contains supplementary material available at 10.1186/s12879-023-08390-x.

## Article Summary

### Article focus

This is the first study that evaluated the periprosthetic environment microbiome in PJI patients after total joint arthroplasty and its potential correlation with inflammation.

### Key messages

We built a “typing system” based on the detected microbiota, and different microbiota types are related to different clinical characteristics.

### Strengths and limitations

This study identified the periprosthetic microbiota differences between PJI and non-PJI patients. The periprosthetic microbiota can be classified into four types generally: “*Staphylococcus* type,” “*Escherichia* type,” “*Cutibacterium* type,” and “*Pseudomonas* type.”

The relatively small sample size in this study is a limitation.

## Introduction

Periprosthetic joint infection (PJI) is one of the most serious complications after total joint arthroplasty (TJA) and often indicates disastrous outcomes [1]. However, with the increasing number of total joint arthroplasty cases, the incidence of PJI is not going to decrease. In the UK and USA, about 800,000 joint arthroplasties are done annually, with projections to greater than 4 million by 2030 [1]. The annual incidence of PJI is estimated at 1% after hip arthroplasties and ranges between 1 and 2% after knee arthroplasties [1]. Kapadia et al. noted a cost of €95,000 per PJI case, which is five times higher than that for primary arthroplasty [1]. Moreover, PJI patients have less satisfaction with their procedure, only up to 23% are satisfied, and 18% report complete dissatisfaction. Therefore, the projected increase in PJI procedures places a huge burden on patients, clinicians, and the worldwide healthcare system [1, 2].

PJI diagnosis is challenging and identifying the corresponding pathogens is the management core in the diagnosis and treatment of PJI. Traditionally, PJI pathogens have been identified by synovial fluid and periprosthetic tissue culture. However, the false-negative rate of culture is about 20–30% for various reasons such as biofilm formation, inert pathogens, inappropriate culture medium, and antibiotic administration [3]. In recent years, to increase the detection rate of PJI pathogens, some molecular methods with high sensitivity such as multiple PCR, untargeted metagenomic next-generation sequencing (mNGS), and targeted next-generation sequencing (NGS) have been used in PJI diagnosis [4–18]. These methods are used to detect pathogens and they often reveal more pathogens than clinical cultures do. In addition, by culturing the sonication fluid of the joint prosthesis retrieved during revisions, the detection rate of polymicrobial PJI also increased [19]. Moreover, studies have found that the discordant rate between preoperative synovial fluid culture and intraoperative synovial fluid culture was about 20% and the culture of specific pathogens such as *E. coli* and *Streptococcus*spp. also indicated higher risks of polymicrobial PJI [20, 21]. These studies not only suggested the potential clinical underestimation of polymicrobial PJI but indicated the existence of heterogeneous polymicrobial microbiota in the periprosthetic environment after TJA. However, to our knowledge, few studies have comprehensively studied the heterogeneous microbiota in this environment [22].

In some patients considered to be aseptic loosening clinically, several inert bacteria such *P. acnes*were shown by mNGS, PCR, prolonged culture time, and the culture of sonication fluid [8, 9, 12]. These phenomena challenge the current notion of the periprosthetic environment as sterile in non-PJI joints after TJA and indicate that microbiota exist in the periprosthetic joint environment despite no significant clinical infection signs. However, most published studies have mainly focused on the suitability of these methods for the diagnosis of pathogens in individual suspected PJI cases [5, 9, 10, 14]. There is no study focusing on the microbiota signature in patients with suspected PJI. In addition, some studies indicated that NGS can be used to detect PJI but the interpretation methods for the results were arbitrary and their diagnostic values were controversial [5, 10]. The major reason can be lack of knowledge about the “relatively normal” periprosthetic microbiota in patients with suspected PJI and ignorance of the microbiota may potentially cause over-treatment because of the high resolution of mNGS to identify pathogens. Hence, a better understanding of the compositional changes in microbiota is necessary so that the role of pathogens in PJI can be further clarified and the bioinformatics pipeline used in interpreting mNGS results can be designed better.

Therefore, we performed a prospective study to shed light on the features of the microbial community in the periprosthetic joint environment after TJA in patients with suspected PJI. Using mNGS to detect microorganisms in the environment surrounding the prosthesis of the revision patient, a classification system based on these major differential microorganisms was established by RandomForest sampling of the major differential microorganisms between PJI and non-PJI. Moreover, our study was also designed to evaluate the specific signatures of the microbiota detected by mNGS in the joint fluid, with PJI confirmed by conventional tests.

## Materials and methods

### Study design

The approval of the Institutional Review Board and the local Ethics Committee were obtained prior to the commencement of this study and then the study was performed in a tertiary joint center. The patients with suspected joint infection with enough synovial fluid for mNGS and standard diagnostic tests were recruited prospectively for this study.

### The definition of patients suspected of having PJI

Based on the institutional protocol, PJI was considered when a patient had one of the following signs or symptoms after TJA.

1. Acute or persistent pain at rest, swelling, redness, or warmth around the joints;
2. Elevated ESR or CRP level; or
3. Implant failure within 5 years after total joint arthroplasty without any reasonable explanation.

**Fig. 1:**
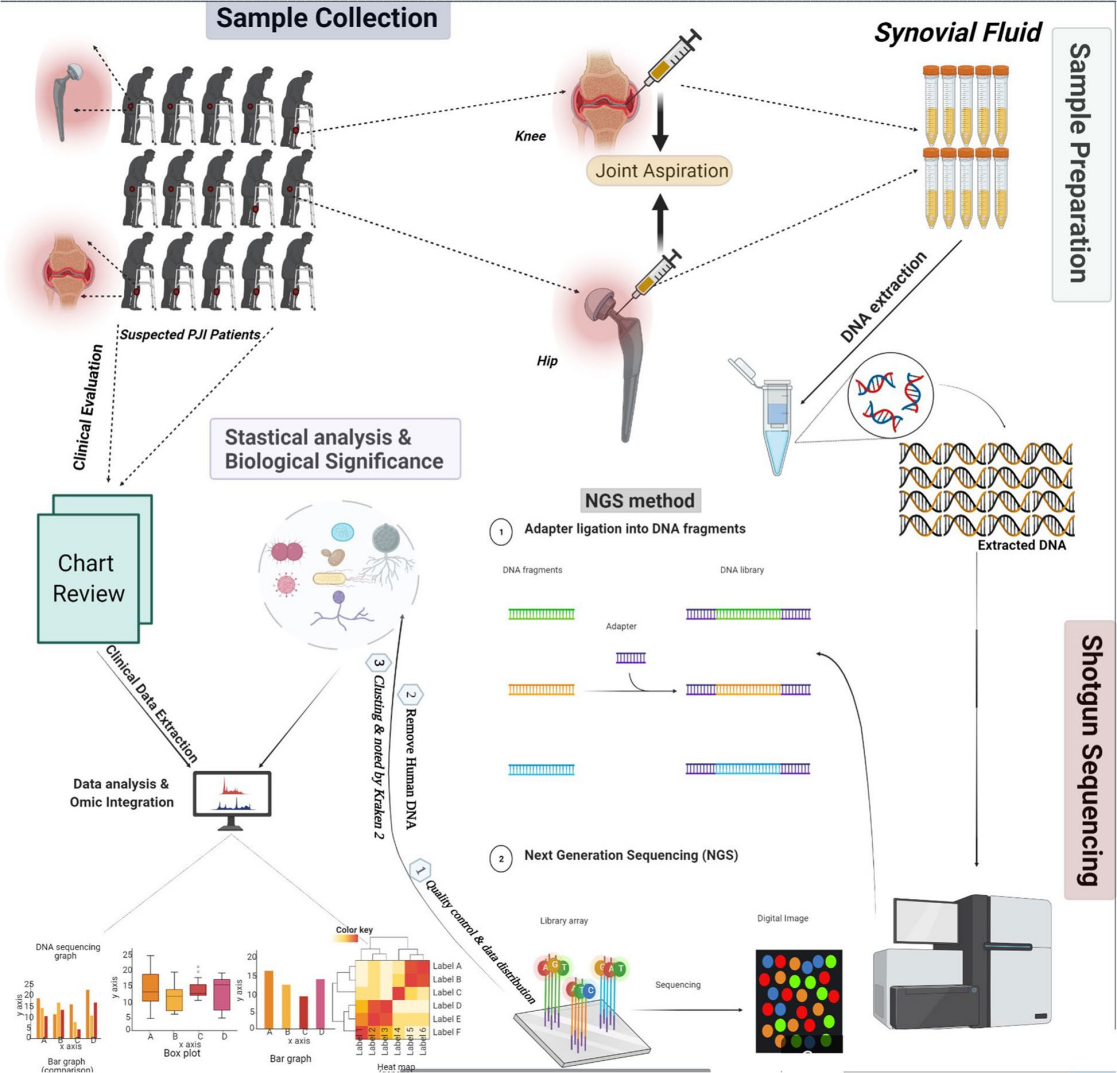
The summary of this study design

All patients provided written informed consent before mNGS tests. In this study, the mNGS lab was blinded to the synovial fluid culture results. Between April 2019 and Match 2020, a total of 77 patients were included in this study. Joint aspiration was performed by two experienced surgeons to obtain synovial fluid for analysis [23]. The obtained joint fluid was sent for mNGS if the patients agreed to participate in the study. The study design is summarized in Fig. 1.

### The PJI diagnosis protocol and the definition of groups

In our institution, ESR and serum CRP was used to screen for PJI. Preoperative joint aspiration was performed in these patients to obtain synovial fluid and then the diagnostic workup was initiated. The methods of preoperative joint aspiration were previously reported by Li et al., and synovial fluid was obtained before the surgeries for joint infection [23]. After joint aspiration, the collected synovial fluid was shipped for mNGS testing, leukocyte esterase (LE) test, synovial fluid analysis (WBC count, polymorphonuclear neutrophils (PMN%)), and anaerobic and aerobic bacterial cultures and fungal culture within 3 h. If PJI can’t be diagnosed based on the 2014 Musculoskeletal Infection Society (MSIS) criteria before joint revisions, intraoperative tissue histology was performed when the joint was revised. Besides, the synovial fluid and 5 periprosthetic tissues obtained during revisions were also sent for cultures during revisions. If PJI was diagnosed based on the 2014 MSIS criteria but no pathogens were identified by cultures (synovial fluid and periprosthetic tissues), the culture-negative PJI was diagnosed. In this study, the diagnosis of PJI was based on the 2014 MSIS criteria.

The patients included in this study were divided into three groups based on the following criteria:

1. Culture-positive PJI group: PJI was diagnosed based on the 2014 MSIS criteria and the pathogens were identified by clinical culture.
2. Culture-negative PJI group: PJI was diagnosed based on the 2014 MSIS criteria but the pathogens were not identified by clinical cultures.
3. Non-PJI group: This group was composed of patients who underwent one-stage aseptic revisions. The one-stage revisions did not fail because of any infection or aseptic reasons such as PJI, loosening, mechanical complications within at least 6-month follow-up after one-stage revision.

### Microbiological cultures

The obtained synovial fluid (> 1 ml) was injected into a BacT/ALERT FA fastidious antimicrobial neutralization (FAN) bottle (BioMerieux) for anaerobic bacterial culture and a BacT/ALERT PF Pediatric FAN (BioMerieux) bottle for aerobic bacterial and fungal culture. Each bottle was incubated for 2 weeks, and VITEK-MS (BioMerieux) was used for microorganism identification if pathogens were detected.

If a microorganism was revealed in either the aerobic bottle or anaerobic bottle, the pathogen was recorded as part of the preoperative aspiration results. Antibiotic sensitivity tests were performed by disk diffusion according to laboratory standard protocols. In addition, two to five different intraoperative periprosthetic tissues were also sent for microbiological cultures during revisions.

### Metagenomic next-generation sequencing and analysis

Volumes of at least 0.5 mL samples were collected from the subjects. To improve the efficiency of pathogen detection, the samples were first enriched in small solutions (~ 200 μL) and centrifuged at 15,000 × *g* for 10 min at 4 ℃. Then 200 μL of enrichment solution was used for nucleic acid extraction and purification with a nucleic acid extraction kit combined with magnetic beads (Sagene, Guangzhou, China); the magnetic bead method had been optimized, as compared with the precipitation method and adsorption column method (Sagene, Guangzhou, China). The metagenomic library was respectively constructed according to the protocol of the library construction kit, Nextera XT (Illumina, USA). The extracted DNA was first divided into ~ 300 bp fragments followed by the addition of different index sequences. During sequencing, no specific DNA fragments were amplified. The library size and quantification were analyzed using an Aglient 2100 bioanalyzer system, and the accurate quantification was detected by qPCR (Bio-Rad CFX96, USA). After the libraries were mixed in equal amounts, high-throughput sequencing was performed on the Illumina Nextseq 550 DX sequencing platform (sequencing strategy: PE150), an FDA-approved sequencer. During the process of library construction and sequencing, no target probes were spiked into the DNA library to capture specific DNA fragments in the microorganisms. In addition, a negative control group was used to eliminate possible DNA contamination during library construction and sequencing.

### Statistical analysis

After the sequencing was finished, raw data were filtered by FastQC software, including removing the reads containing the sequencing adapters, the reads containing more than 10% N, and the low-quality reads containing less than 50% of low-quality bases (Q-value ≤ 10). The remaining reads were used for next-step analysis. Human-related reads were removed by aligning with the human genome reference sequence (version: GRCh38), using BWA (http://bio-bwa.sourceforge.net/) software, and then a proprietary microbial pathogen database (Kraken2/Bracken) was used for analysis to obtain the identification and quantitative results of pathogenic microorganisms. The microorganisms reference databases were downloaded from NCBI (ftp://ncbi.nlm.nih.gov/genom es/). Then, possible DNA contamination in the reagent was filtered by SourceTracker [24]. Relative abundance data were profiled in comparison analysis, and clinical relevance analysis was mainly based on R. Alpha-diversity was calculated based on Shannon indexes at the species level. Bray–Curtis distance was used to perform principal coordinate analysis (PCA) based on the species levels. The Circos graph (www.circos.ca) was plotted based on the top 10 genera with the highest relative abundance in the microbiota. We used the RandomForest bag (R, version: 4.1.1) to build a RandomForest model and extracted the top five major features (multiple displacement amplification, MDA method) and the “typing system” was built based on these major microorganisms. Then the abundance of these major microorganisms was translated into relative abundance based on these five microorganisms for further analysis. We used Corrplot for interactively analyzing microbiome data and clinical data. The correlation was evaluated by the Wilcoxon rank-sum test. Clustering was dependent on the Ward distance.

## Results

### The study design and the characteristics of patients included in this study

There were 42 and 35 patients in the PJI group and non-PJI group, respectively. The median age in the PJI group and non-PJI group was 65 years and 67 years, respectively. In this study, only hip and knee joints were included. There were 30 and 26 knee joints in the PJI group and non-PJI group, respectively. The details of the patients included in this study are summarized in Table 1.

**Table 1:** Characteristics of patients included in this study

	PJI Group (*n* = 42)	Non-PJI group (*n* = 35)	*P* value
Age (years)	65 (30, 82)	67 (45,78)	0.742
Sex			
Male sex-no. (%)	17, 40.5%	12, 34.3%	0.577
Female sex-no. (%)	25, 59.5%	23, 65.7	0.577
Affected joint			
Knee-no. (%)	30,7 1.4%	26, 7 4.3%	0.779
Hip-no. (%)	12, 28.6%	9, 25.7%	0.779
Height(m)	1.6 (1.31, 1.86)	1.61 (1.48, 1.86)	0.917
Weight(kg)	68 (42.8, 93)	64 (45, 90)	0.073
BMI (kg/m^2^)	26.17 (18.04, 31.43)	24.03 (16.52, 30.07)	0.008
Acute phase^a^	7,1 6.7%	2, 5.7%	0.136
Synovial fluid WBC (cells/ul)	15,798 (18, 150,000)	685 (0, 43,700)	0.002
Synovial fluid PMN%	91 (16, 99)	27 (0, 97)	< 0.0001
Serum CRP (mg/dl)	2.16 (0.1, 14.59)	0.67 (0.09, 12.55)	0.001
ESR (mm/hr)	59 (4, 123)	16.5 (2,88)	< 0.0001
Negative culture-no. (%)	14, 33.3%	32, 91.4%	< 0.0001
The presence of sinus	5, 11.9%	0	0.043

The values are given as medians with the range in the parentheses

^a^Acute phase: first postoperative month// < 3 weeks of symptoms

### The distribution and diversity of the periprosthetic microbiota

**Fig. 2:**
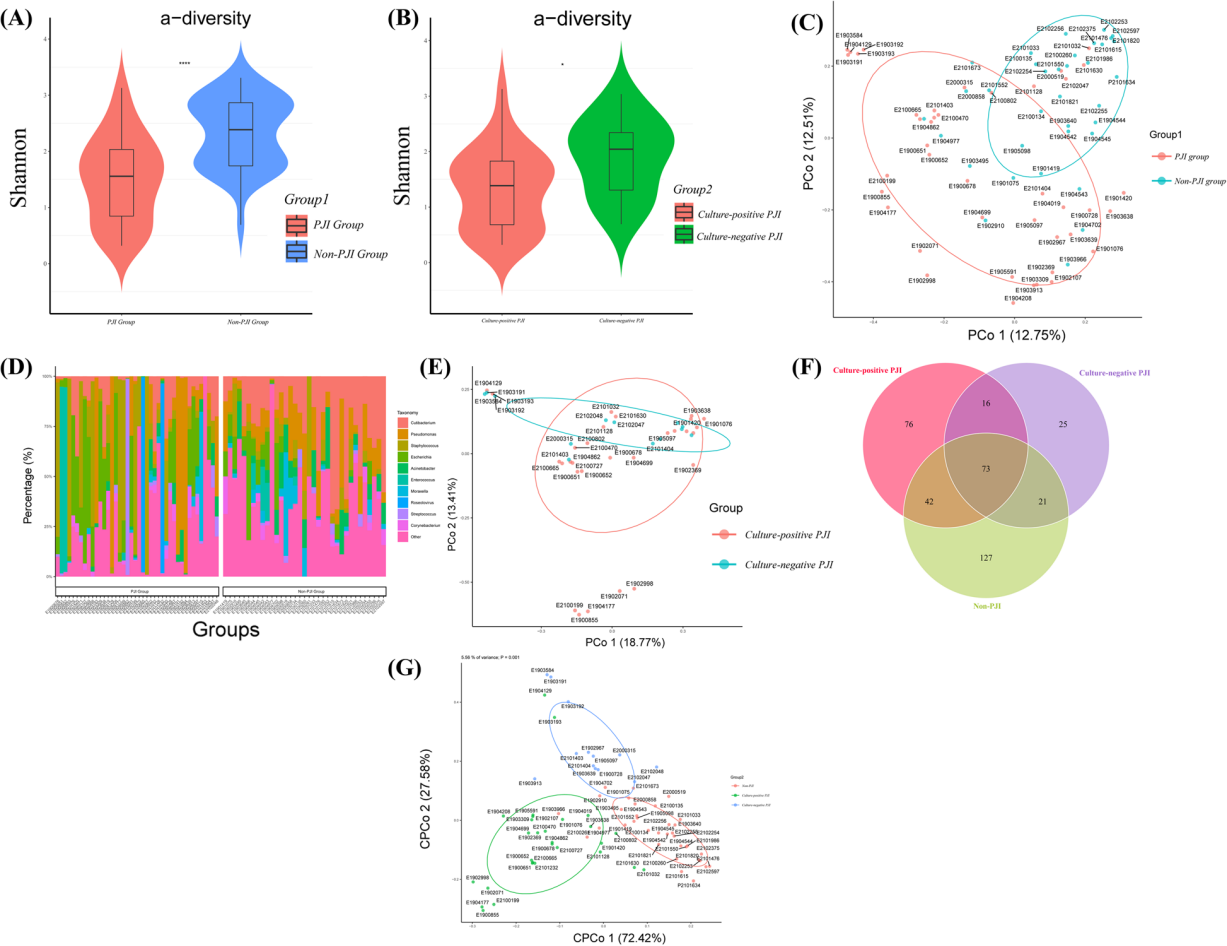
PJI patients and non-PJI patients harbor a distinct microbiome. **a** Boxplots of microbial Alpha-diversity (Shannon's index) for the synovial fluid from PJI patients and non-PJI patients. **b** Boxplots of microbial Alpha-diversity (Shannon’s index) for the synovial fluid from culture-positive PJI patients and culture-negative PJI patients. **c** PCoA plot of the microbiota using Bray–Curtis distance metric of Beta-diversity in the synovial fluid of PJI patients and non-PJI patients. **d** Genus-level distribution of the microbiota in the PJI patients and Non-PJI patients. **e** PCoA plot of the microbiota using Bray–Curtis distance metric of Beta-diversity in the synovial fluid of culture-positive PJI patients and culture-negative PJI patients. **F** The Venn plot shows the overlaps among the non-PJI group, culture-positive PJI group, and culture-negative PJI groups at the specie level. **g** CPCoA plot of the microbiota using Bray-Curtis distance metric of Beta-diversity in the synovial fluid of culture-negative PJI patients, culture-positive PJI patients, and non-PJI patients

The characterization of periprosthetic microbiota was different between the PJI group and the non-PJI group. As shown in Fig. 2, Shannon’s index and Simpson diversity index in the PJI group were lower than that in the non-PJI group. Similarly, as shown in the PCA analysis (Fig. 2c), the beta-diversity in these two groups was significantly different, and the samples can be classified into two groups based on the Bray-Curtis distance. Some pathogens in the PJI group were more likely to show higher abundance than that in the non-PJI group, such as *S. aureus*. However, some pathogens in the PJI group had a lower abundance than that in the non-PJI group, such as *P. acnes*. The distribution of microorganisms in PJI and non-PJI groups is summarized in Fig. 2. Generally, there were some overlaps in microbiomes between the PJI group and non-PJI groups. The PJI cohort was then divided into culture-positive PJI and culture-negative PJI based on the clinical culture results, and Shannon’s index in the culture-negative PJI group was higher than that in the culture-positive PJI group.

### The construction of a “typing system” of periprosthetic microbiota based on RandomForest model

**Fig. 3:**
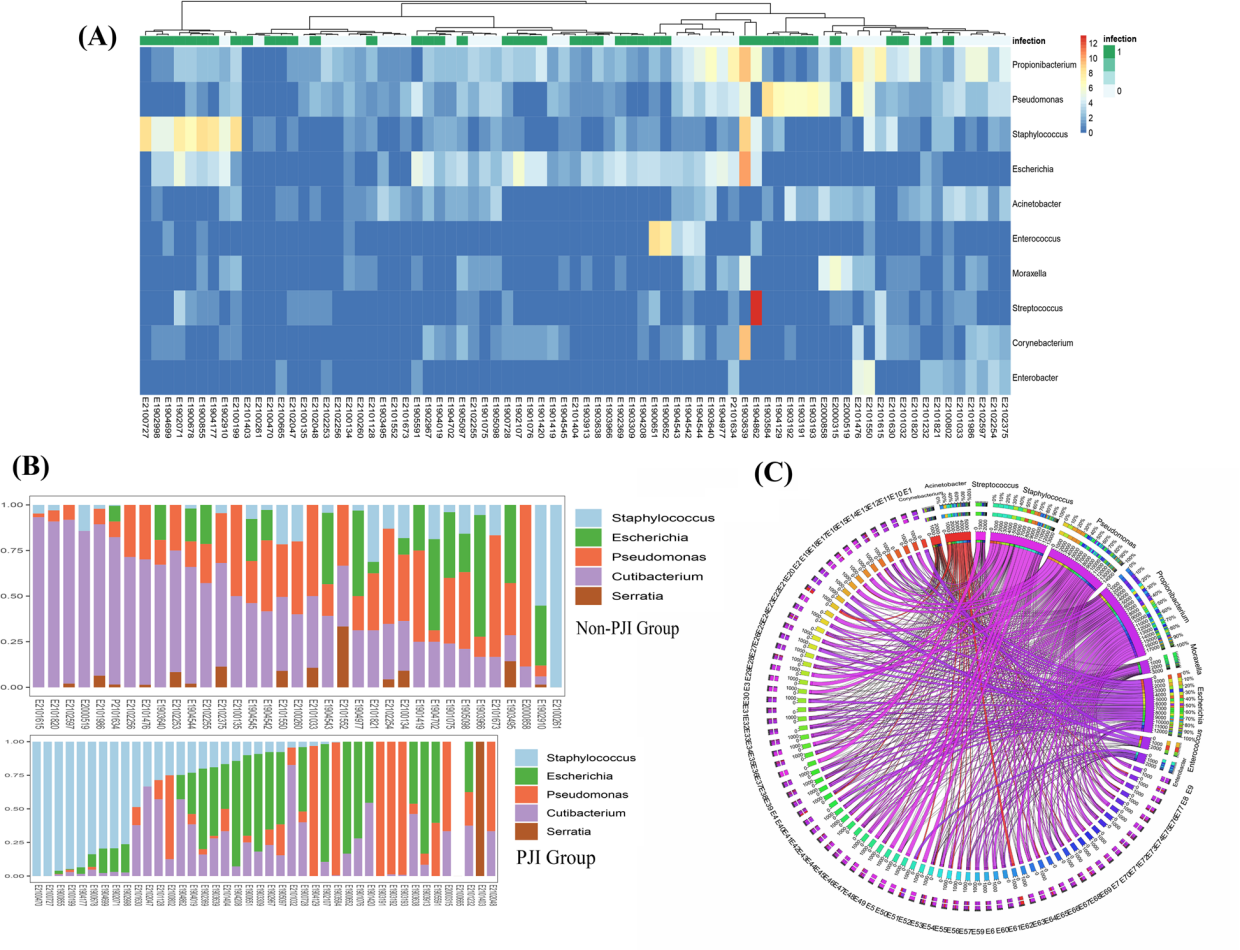
The genus-level distribution of the microbiota in the PJI patients and non-PJI patients. **A** Heatmap showing the relative abundance of the microbiota (top 10) clustered by the Ward method based on candidate microbiota. **B** Bar plot denoting the relative abundance of five bacteria selected by the RandomForest model at the genus level for each sample. The x-axis indicates the number of patients with PJI or non-PJI. The y-axis represents the detection ratio of target microbiomes based on the average relative abundance. **C** The Circos plot shows the distribution of the relative abundance of the top 10 microorganisms within each sample at the species level

The clustering of microbiota distribution suggested that there can be different “microbiota types” in the periprosthetic environment after TJA. We extracted the major microorganisms from the microbiota based on the RandomForest model (Supplementary Fig. 1). According to the major microorganisms of the microbiota extracted from the RandomForest model and the abundance of these microorganisms detected in the periprosthetic microbiota, the microbiota can be classified into four types corresponding to different clinical signatures:

1. *Cutibacterium* type: The details of the microbiota distribution in this type are shown in Fig. 3. In this type, the specific highly abundant microorganisms were *Cutibacterium* spp., and the clinical diagnosis of this type was often non-PJI, indicating that the clinical signs of infection in patients with this type (ESR, CRP) were not obvious.
2. *Staphylococcus* type: In this type, the highly abundant microorganisms were *Staphylococcus* spp*.,* and corresponding patients were more likely to be diagnosed as having PJI. Clinically, in this type (a total of 11 cases), 10 cases were diagnosed as PJI based on the 2014 MSIS criteria.
3. *Escherichia* type: In this type, the most abundant microorganisms were *Escherichia* spp., and the corresponding patients were likely to be diagnosed as having PJI.
4. *Pseudomonas* type: In this type, the most abundant microorganisms were *Pseudomonas* spp., and the corresponding patients were likely to be diagnosed as having PJI.

To further show the distribution of these microorganisms within each sample, a Circos plot was built at the level of genus (Fig. 3) according to the top 10 most abundant microorganisms. The proportions of different periprosthetic microbiome types in PJI and non-PJI groups are shown in Table 2.

**Table 2:** The proportion of different periprosthetic microbiome types in PJI and non-PJI groups

	Cutibacterium type	Staphylococcus type	Escherichia type	Pseudomonas type	Cutibacterium + Pseudomonas type^a^
Non-PJI group, n (%)	17 (51.5%)	1 (3%)	5 (15.2%)	1 (3%)	9 (27.3%)
Culture-positive PJI group, n (%)	3 (11.5%)	8 (30.8%)	11 (42.3%)	2 (7.7%)	2 (7.7%)
Culture-negative PJI group, n (%)	0	2, (15.4%)	5 (38.5%)	3 (23.1%)	3 (23.1%)

^a^In this type, the abundance of Cutibacterium spp. and Pseudomonas spp. are comparable

### The clinical data on different types of microbiota in the periprosthetic environment

**Fig. 4:**
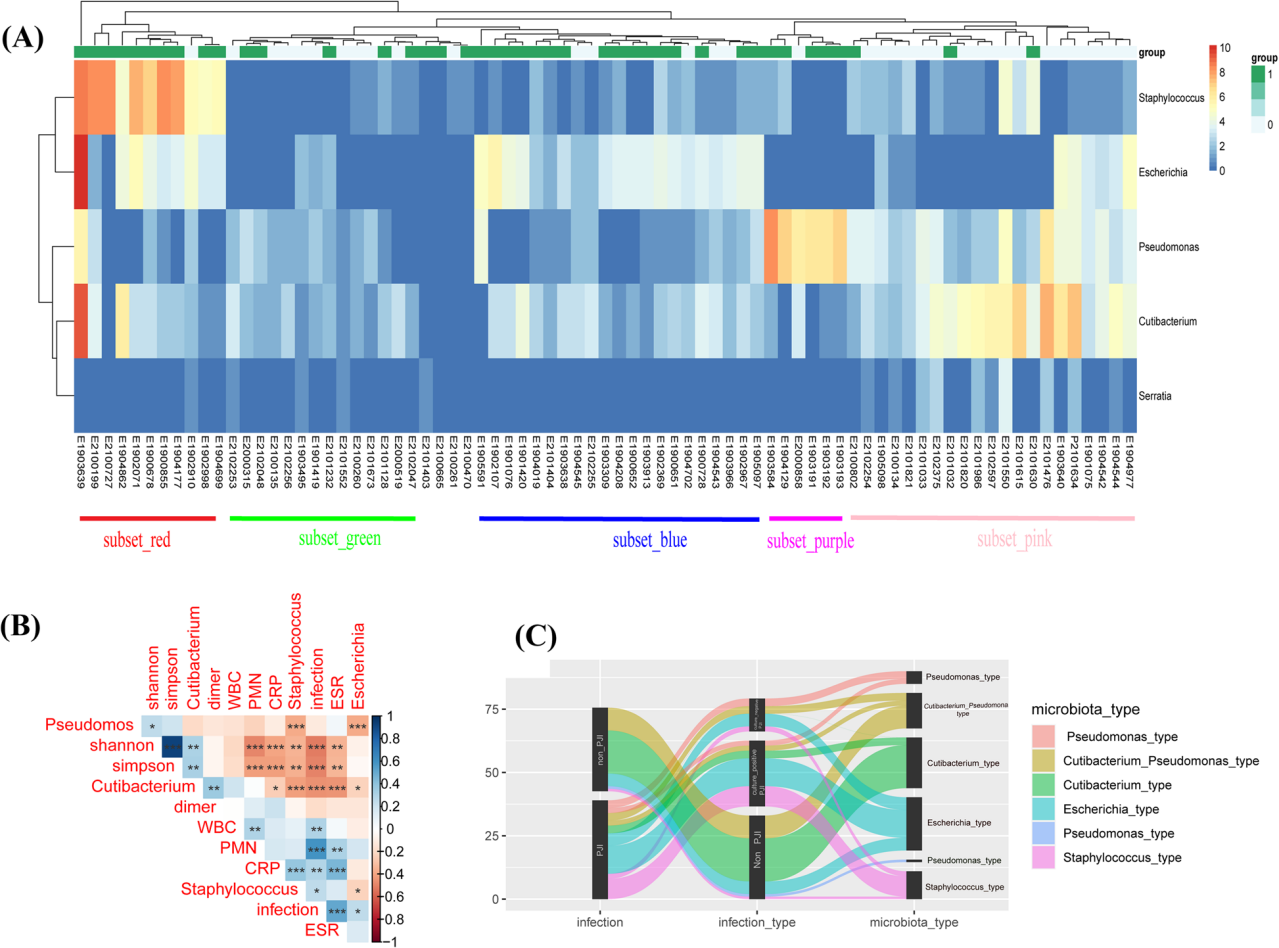
The relationship between different microbiota types and clinical data. **A** Heatmap denoting the relative abundance of five bacteria selected by the RandomForest model at the genus level for every five subsets (subset_red, subset _green, subset _blue, subset _purple, subset _pink) clustered by Ward’s method based on candidate microbiota in the synovial fluid of patients. **B** The heatmap shows the relationship between the clinical characteristics and microbiota characteristics. *P < 0.05; *P < 0.01; ***P < 0.001. **C** Synovial fluid with a predominance of different microorganisms has different clinical diagnoses. Sankey’s diagram shows the relationship between the types based on microbiota and clinical diagnosis. Shannon: Shannon–Wiener diversity Index. Simpson: Simpson’s diversity index

To further classify the association between the microbiota and clinical signatures, we integrated the clinical omics and the abundance data about these microorganisms. The abundance of *Staphylococcus* spp. is positively correlated with the levels of ESR, serum CRP, and the presence of clinical PJI. However, the abundance of *Cutibacterium* spp. is negatively correlated with the levels of ESR, CRP, the presence of clinical PJI, the abundance of *Staphylococcus* spp., and *Escherichia* spp. The details about the relationships are summarized in the cluster rectangles (Fig. 4 and Table 3). In addition, the relationship between different types of periprosthetic microbiota and clinical diagnostic results is shown in the Sankey plot (Fig. 4). As shown in this figure, the “*Staphylococcus* type” was more likely to appear in the infection group compared to other types while the “*Cutibacterium* type” was more likely to appear in the non-infection group.

**Table 3:** Clinicopathological characteristics of different microbiota types

Clinical indicators	Staphylococcus type	Cutibacterium type	Pseudomonas type	Escherichia type	Cutibacterium type + Pseudomonas type
Blood					
CRP^a^ (mg/dl)	5.53 ± 5.6	1.67 ± 2.39	4.25 ± 3.19	1.935 ± 1.80	2.36 ± 3.296
ESR^a^ (mm/hr)	53.18 ± 38.21	16.7 ± 19.38	67.5 ± 36.35	52.33 ± 29.22	30.5 ± 22.5
Synovial fluid					
PMN%^a^	79.2 ± 26.3	49.9 ± 38.2	74.67 ± 32.12	78.5 ± 29.14	65.36 ± 35.5
WBC^a^ count (cells/ul)	27,517 ± 42,426	14,741 ± 30,373	16,930 ± 17,613	15,418 ± 18,612	31,360 ± 47,368
Shannon index^a^	1.19 ± 0.80	2.59 ± 0.57	1.65 ± 0.68	1.86 ± 0.73	1.83 ± 0.48

^a^The values are given as means ± sd

### External validation of the typing profile and its correlation with inflammation

To further clarify the dedicated and specific correlation between the ‘typing profile’ and inflammation. External verification was performed in the other cohort (including 168 patients). In this cohort, the metagenomic data generated from 168 patients, were obtained from NCBI (PRJNA436717).

**Fig. 5:**
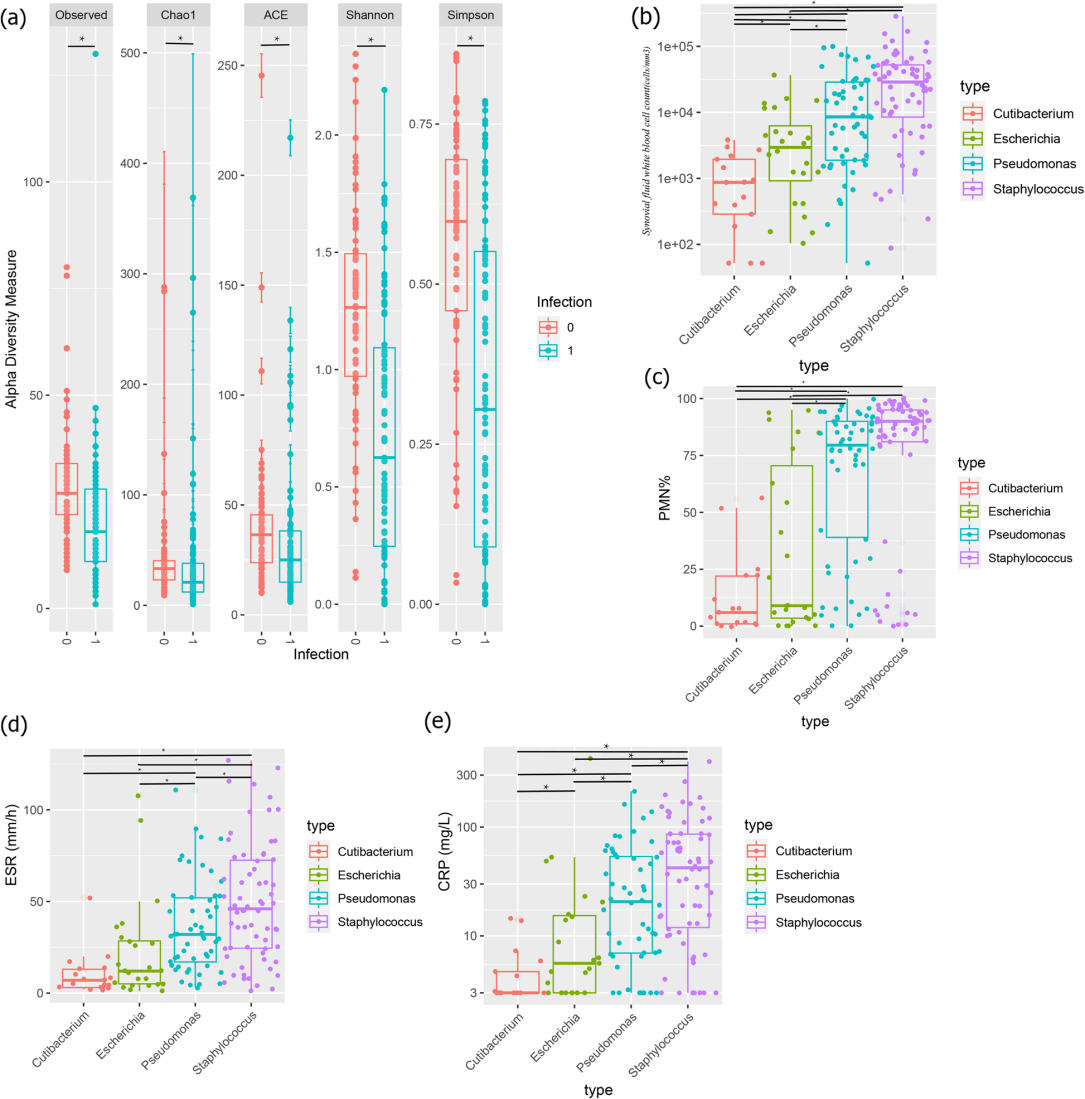
Metagenomic analysis of the correlation between periprosthetic microbiota structure and inflammation. **a**The alpha-diversity in the non-PJI group was significantly higher than that in the PJI group. (Chao1: Chao1 richness estimator; ACE: ACE estimator; Shannon: Shannon Wiener index; Simpson: Simpson diversity index). *P < 0.05 (Mann–Whitney U-test). The levels of synovial fluid WBC count (**b**), synovial fluid PMN% (**c**), ESR (**d**), serum C-reactive protein (**e**) in patients with those four types of periprosthetic microbiota. Box plots reflect median and IQRs with whiskers bounding non-outlier values. *P < 0.05 (Mann–Whitney U-test)

The microorganism abundance tables of these samples were obtained according to the bioinformatic pipeline mentioned in the ‘methods part’ and then, the relative abundance of the “major microorganisms” which were selected in the previous RandomForest model was extracted. We tested the diagnostic accuracy of the pre-built RandomForest model for PJI diagnosis compared to the MSIS criteria in this validation cohort and the AUC of the RandomForest model for PJI diagnosis was 0.81. Besides, the alpha-diversity in the non-PJI group was significantly higher than that in the PJI group (Fig. 5a), which was consistent with the results we obtained in the exploration cohort.

**Fig. 6:**
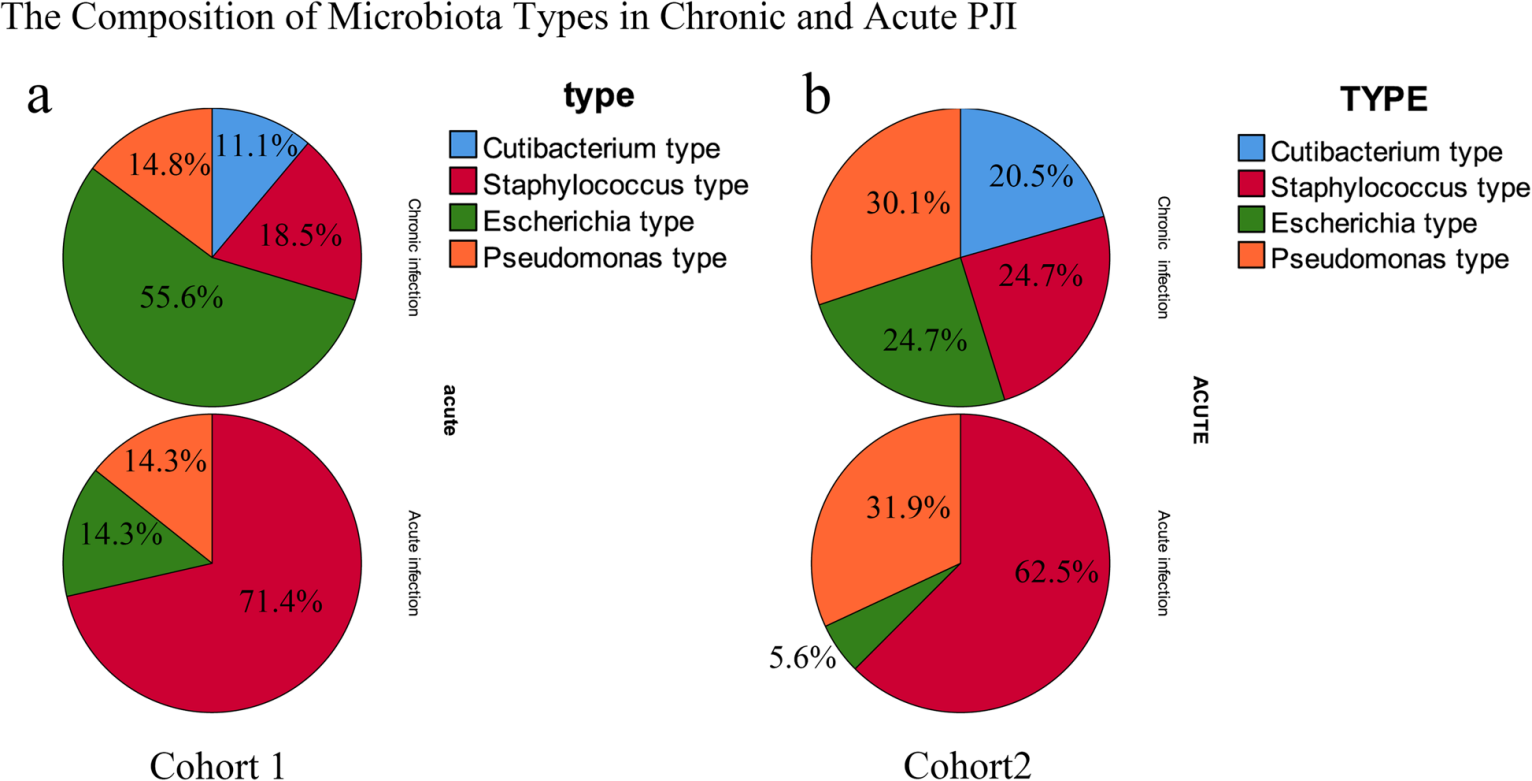
The pie graphs showing the composition of microbiota types in chronic and acute PJI in two cohorts. The patients (77 patients) in cohort1 were recruited by the authors. The cases (168 cases) in cohort 2 were obtained from the public database (PRJNA436717)

After that, the patients in the validation cohort were classified into 4 types based on the relative abundance of the selected ‘major microorganism’: “*Staphylococcus* type,” (71 cases) “*Escherichia* type,”(24 cases) “*Cutibacterium* type,”(17 cases) and “*Pseudomonas* type.”(54 cases). The incidence of PJI in the ‘*Staphylococcus* type’ was 78.9% (56/71), significantly higher than that in the ‘*Cutibacterium* type’ (78.9% vs 0%, *P* < 0.05). Then, we evaluated the levels of host inflammatory responses to these different types of periprosthetic microbiota. We found that the levels of ESR, serum CRP and synovial fluid WBC count, synovial PMN% in the patients with ‘*Staphylococcus* type’ were significantly higher than that in the patients with ‘*Pseudomonas* type’, followed by ‘*Escherichia* type’ and ‘*Cutibacterium* type’ (Fig. 5bcde). The intensity of the inflammatory response shows a stepwise distribution in patients with different types of periprosthetic microbiota. The details were shown in Fig. 5 and Table 4. Moreover, the percentage of cases with “*Staphylococcus* type” in the acute PJI group was higher than that in the chronic PJI group (Cohort1: 71.4% vs 18.5%; *P* = 0.043; Cohort2: 62.4% vs 24.7%; *P* < 0.0001). However, the percentages of cases with “*Cutibacterium* type” in the acute PJI group was lower than that in the chronic PJI group (Cohort1: 11.1% vs 0; *P* = 0.036; Cohort2: 20.5% vs 0; *P* < 0.0001). These data indicated that chronic and acute PJI were of different microbiota types, suggesting different microbiota composition between acute and chronic PJI (Fig. 6).

**Table 4:** Clinicopathological characteristics of different microbiota types in the validation cohort

Clinical inflammatory indicators	Staphylococcus type	Pseudomonas type	Escherichia type	Cutibacterium type
Blood				
ESR^a^ (mm/h)	49.86 ± 33.4	37.3 ± 25.6	25.95 ± 29.9	6.55 ± 4.8
CRP^a^ (mg/L)	66.2 ± 77.9	40.4 ± 46.3	33.7 ± 96.2	5.6 ± 4.4
Synovial fluid				
Synovial fluid WBC^a^ (cells/mm3)	43,618 ± 53,990	18,693 ± 24,545	6443 ± 8817	970 ± 949.4
Synovial fluid PMN%^a^	75.5 ± 33.9	67.9 ± 30.7	34.4 ± 38.4	4 ± 16.98

^a^The values are given as means ± sd

## Discussion

This study systematically examined the characterization of the periprosthetic environment microbiome in 77 patients with suspected PJI after TJA. Our study suggested that the characterization of the periprosthetic environment microbiome was significantly different between the PJI group and the non-PJI group. Results show that the diversity of microbiota in the PJI group was significantly lower than that in the non-PJI group. In general, bacterial biodiversity can be a relatively good marker in some “populated” environments [25]. From this point of view, the increase of bacterial biodiversity can potentially indicate a relatively “healthier” periprosthetic environment. Moreover, based on the features extraction by the RandomForest model, the periprosthetic microbiota in patients after TJA can be classified into four different types: *Cutibacterium* type, *Staphylococcus* type, *Escherichia* type, and *Pseudomonas* type. In the patients with the *Cutibacterium* type, the clinical signs and symptoms were relatively “weak” and these patients were more likely to be classified clinically as non-PJI patients based on the 2014 MSIS criteria compared to the other types. Conversely, in the “*Staphylococcus* type”, PJI was more likely to be diagnosed after undergoing septic revisions. *Staphylococcus* spp., including *S. aureus* and coagulation-negative *staphylococcus*, are the leading causes of PJI in clinical practice. *E. coli*is a gram-negative pathogen and the main gram-negative bacteria causing PJI [1]. As the literature reports, *Cutibacterium*spp. is an emergent pathogen causing a low-grade infection (CRP and other clinical and laboratory parameters are often negative) [12, 13]. In addition, in some presumed aseptic loosening patients, *Cutibacterium* spp. are also isolated by prosthesis sonication fluid culture [22, 26]. *Pseudomonas*spp. are a group of conditionally pathogenic bacteria, commonly found in hospital infections [27, 28].

To our knowledge, this is the first study that evaluated the characterization of the periprosthetic environment microbiome in patients with suspected PJI after TJA. Consistent with clinical findings, some microorganisms of high abundance in the microbiota were more likely to be isolated in clinical cultures [10, 29]. Considering the relatively low abundance of these microorganisms in the non-PJI patients, these microorganisms may reach an ecological “peak” in the development of PJI after TJA followed by obvious clinical PJI symptoms and signs.

To further clarify the microbiota composition in suspected PJI patients after TJA, we extracted the major organisms contributing most to the microbiota composition to depict the microbial community and established a “typing system” based on the RandomForest model [30]. Generally, the microbiota composition in suspected PJI patients can be grouped into four different types: *Cutibacterium* type, *Staphylococcus* type, *Escherichia* type, and *Pseudomonas* type. In the patients of the *Cutibacterium* type and *Pseudomonas* type, PJI is not likely to be diagnosed as compared with those of the *Staphylococcus* type and *Escherichia* type. In addition, the levels of some clinical inflammatory indicators (serum ESR, CRP, and synovial fluid WBC) were increased with the increasing abundance of *Staphylococcus* spp. and decreased with the decreasing abundance of *Cutibacterium* spp. It also indicated that the inflammatory response was more intense in the *Staphylococcus* type than that in the *Cutibacterium* type. Clinically, some studies detected *Cutibacterium*spp. in the sonication fluid of prostheses retrieved from assumed aseptic loosening cases [22, 26, 31], suggesting that *Cutibacterium* spp. can be “relatively normal” microorganisms in patients with suspected PJI, and it may not usually cause clinically obvious PJI after TJA because the virulence of *Cutibacterium* spp. is lower than that of *Staphylococcus* spp. However, if *Staphylococcus* spp. become highly abundant in the periprosthetic microbiota, the clinical inflammatory response can be severe because *Staphylococcus* spp. is highly virulent and can cause obvious local and systemic inflammatory responses. *Staphylococcus*spp. is also the most common pathogen that causes PJI [1].

In clinical practice, positive culture and the presence of a sinus communicating with the prosthesis were thought to be the “gold standard” for PJI diagnosis [32–35]. However, culture-negative PJI still accounts for about 20–30% of all PJI in patients [3]. In these patients, molecular diagnostic methods such PCR and mNGS are used to identify the offending pathogens and the use of these techniques is increasing [6, 14, 18]. At present, the interpretation of mNGS sequencing results for PJI diagnosis remains controversial, and diagnostic accuracy is heterogeneous [6, 14, 36, 37]. The major reason can be the lack of knowledge about the microbiota in patients with suspected PJI [22]. Our study explored this problem and showed that PJI is not likely to be diagnosed clinically if another highly abundant microorganism is detected by mNGS, such as *Cutibacterium*spp. This is consistent with previous studies showing that these pathogens are likely to be isolated in the sonication fluid of the prosthesis retrieved during aseptic revisions [13, 19]. In addition, several studies focusing on the utility of RCR in PJI diagnosis also reported similar results [18]. In this study, the microbiota with these special characteristics were classified into *Cutibacterium* type, and corresponding patients had weak inflammatory responses (low levels of synovial fluid WBC count, PMN%, ESR, and serum CRP). Therefore, we hypothesize that these pathogens can be the “relatively native microbiota” that colonize the periprosthetic environment after TJA, and these may not usually cause obvious inflammatory responses or trigger significant septic symptoms. More importantly, these pathogens should be considered when the accurate interpretation pipeline of mNGS results is designed because the misleading interpretation of these pathogens can cause overuse of antibiotics, especially in culture-negative PJI.

In the validation cohort, the results strengthen the conclusion that the microbiota diversity in the non-PJI patients was higher than that in PJI patients. Besides, the corresponding hosts showed obvious different inflammatory responses to different types of periprosthetic microbiota. The host inflammatory response to ‘*Staphylococcus* type’ was significantly more severe than ‘*Pseudomonas* type’, followed by ‘*Escherichia* type’ and ‘*Cutibacterium* type’. From the view of microbiota, the hosts show different inflammatory responses to the microbiota when the periprosthetic environment was dominated by different microorganisms. Clinically, nucleic acid sequence of pathogens could still be detected by mNGS even when the culture results were negative and it suggests that metagenomic can be used as a complementary approach to conventional cultures for PJI diagnosis, just as other pieces of literature reported [5, 10, 38]. Taken together, the composition of microorganisms in the periprosthetic environment is not only related to PJI but also reflects the levels of host inflammation. In other words, the disturbances of the microbiome might affect the development of PJI potentially and their composition may also be promising in monitoring the treatments outcomes of PJI patients or providing a reference for clinical diagnosis and prognosis.

With the increasing use of clinical mNGS for PJI diagnosis, the exploration of periprosthetic microbiota can contribute to the establishment of the bioinformatic pipeline for mNGS results [13, 36, 39, 40]. For example, if the detected periprosthetic microbiome is classified as *Cutibacterium*type and the clinical tests cannot provide strong evidence for PJI, the aseptic loosening is more likely to be considered based on the PJI diagnosis criteria [19, 41]. In addition, prior studies have revealed that some of the cases classified as aseptic may be infectious in origin but either was not investigated or escaped diagnosis of PJI using the available modalities [38, 42]. Taken together, these phenomena suggest the need for further research into the role of *Cutibacterium* in PJI and presumed aseptic loosening. In this study, 17 patients (classified as *Cutibacterium* type) with a clinical diagnosis of aseptic loosening did not develop PJI within postoperative 6-month follow-up. Correspondingly, if the periprosthetic microbiome was classified as the *Staphylococcus* type, PJI can be highly suspected based on the PJI diagnosis criteria, and septic revisions can be an option. In this study, PJI was confirmed by the 2014 MSIS criteria in 10 out of 11 cases of the *Staphylococcus* type. Therefore, the bioinformatic pipeline based on the “typing system” has promising value in helping clinical decision-making.

There are still some limitations in this study. First, the samples were collected in the same hospital, and selection bias was inevitable. Therefore, the classification system proposed in this study still needs to be validated extensively. Second, we studied only the composition of periprosthetic microbiota, and the function of the microbiota was not evaluated in this study because of the relatively low sequencing volume. Our follow-up study will try to interpret microbiota function with the use of metabolomics [36].

Overall, our study sheds light on the composition of the periprosthetic environment microbiome in patients after TJA, and the microbiota could be classified into four types: *Staphylococcus* type, *Escherichia* type, *Cutibacterium* type, and *Pseudomonas* type. Different types correspond to different levels of inflammatory responses and clinical features. This result can be a reference for the design of appropriate mNGS reporting criteria in clinical practice and aid in future studies on the periprosthetic microbiome in patients with PJI.

## Supplementary Information

**Additional file 1: Supplementary figure 1.** The top 30 variables with highest mean decrease accuracyvalues in random forest model.

**Additional file 2: Appendix 2.** The survival curve of the association between microbiota types and clinical outcomes.

## Data Availability

All data and materials were in full compliance with the journal’s policy. And the data were obtained in Department of Orthopedic Surgery, The First Medical Center, Chinese PLA General Hospital. The datasets used and during the current study are available from the corresponding author on reasonable request. The datasets generated and/or analysed during the current study are available in the NGDC repository, [ACCESSION NUMBER: PRJCA016981].

## Abbreviations

TJA: Total joint arthroplasty
PJI: Periprosthetic joint infection
