# Supplementary figures and images for: Characterization of periprosthetic environment microbiome in patients after total joint arthroplasty and its potential correlation with inflammation
Source: BMC Infect Dis. 2023 Jun 22;23:423. doi: 10.1186/s12879-023-08390-x (PMC10286366; doi:10.1186/s12879-023-08390-x)

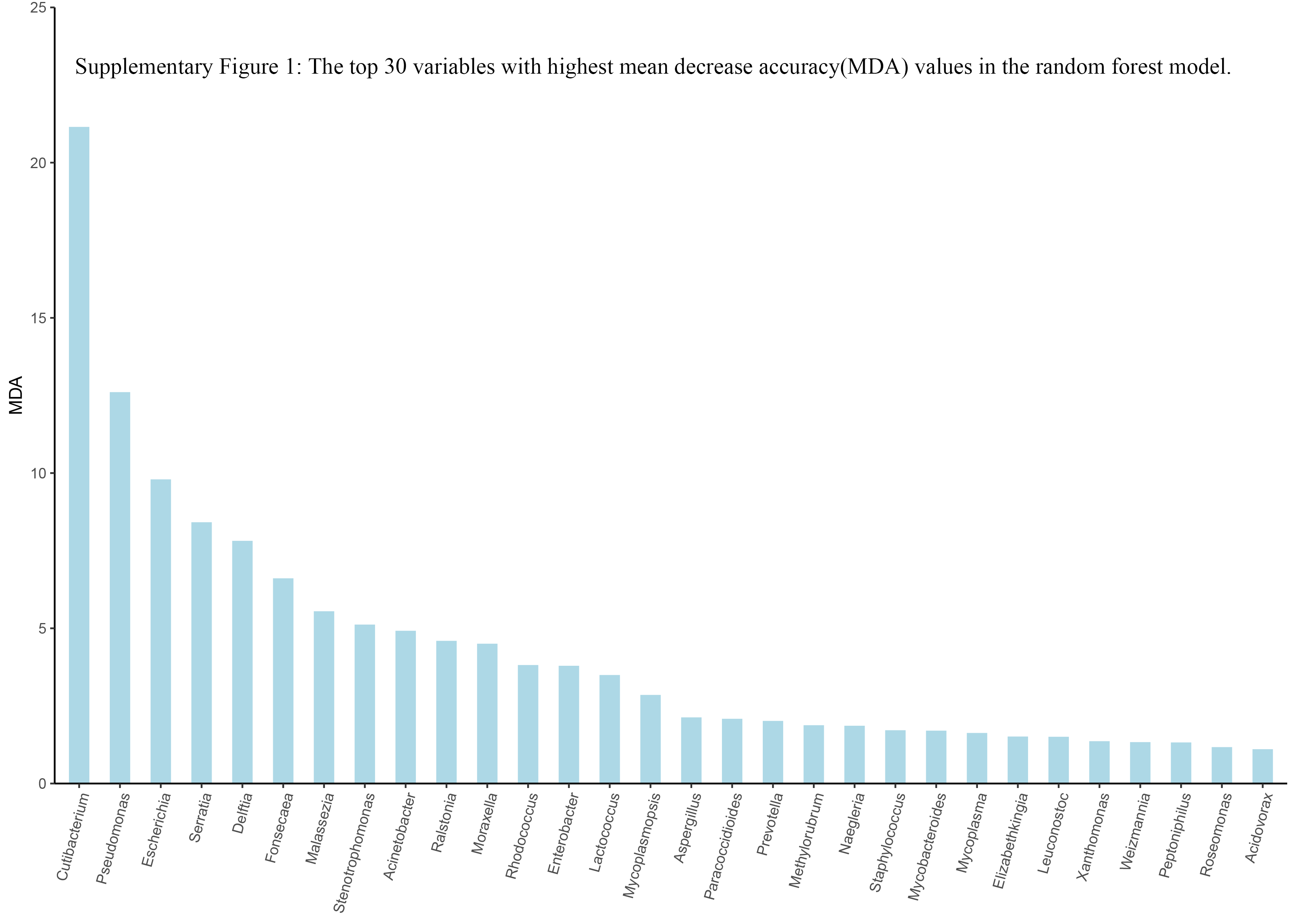

Supplement: Supplementary file 1 — Additional file 1: Supplementary figure 1. The top 30 variables with highest mean decrease accuracyvalues in random forest model. [file 12879_2023_8390_MOESM1_ESM.tif]

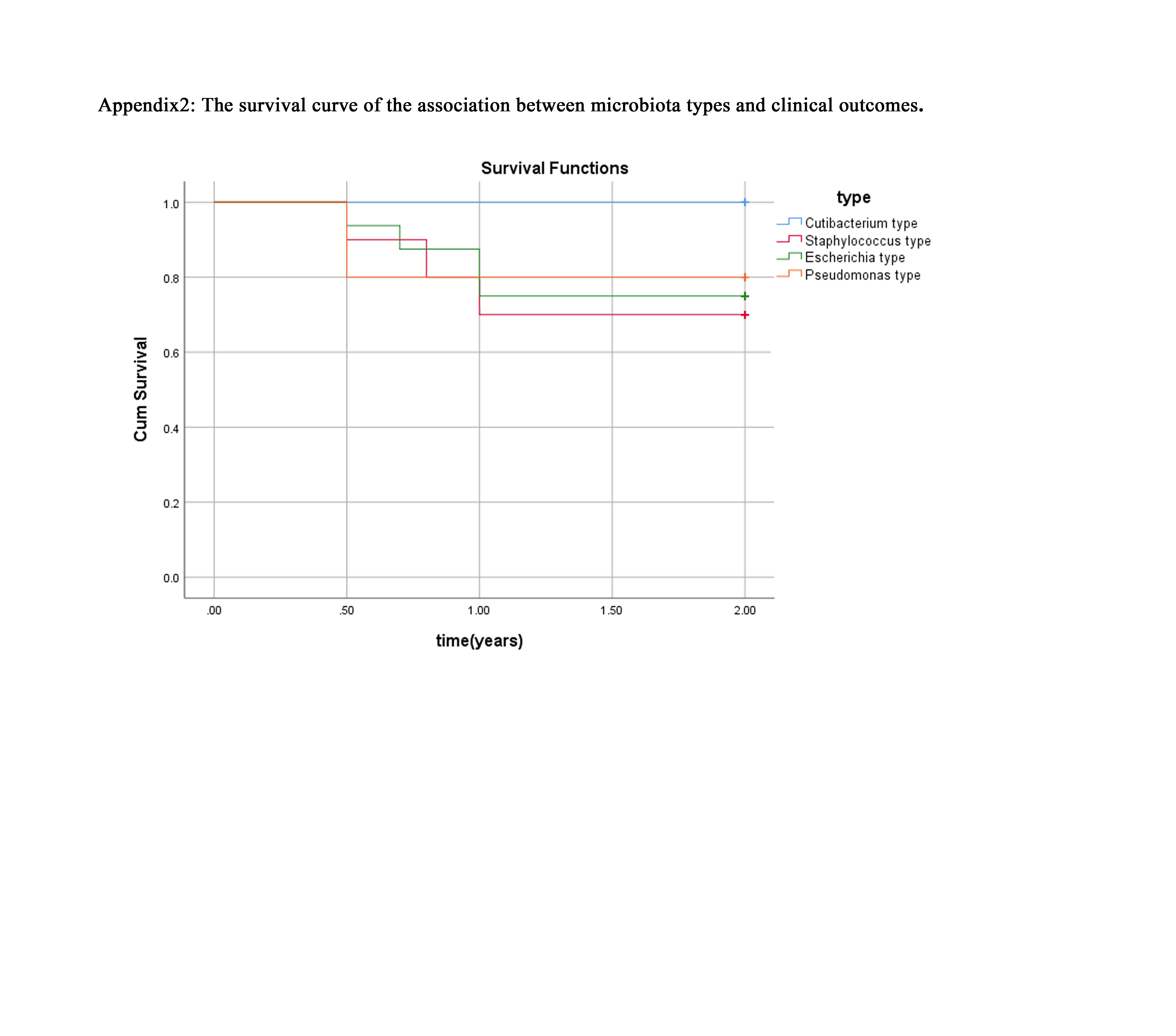

Supplement: Supplementary file 2 — Additional file 2: Appendix 2. The survival curve of the association between microbiota types and clinical outcomes. [file 12879_2023_8390_MOESM2_ESM.png]
